# Automated reminders reduce incarceration for missed court dates: Evidence from a text message experiment

**DOI:** 10.1126/sciadv.adx7483

**Published:** 2025-10-01

**Authors:** Alex Chohlas-Wood, Madison Coots, Joe Nudell, Julian Nyarko, Emma Brunskill, Todd Rogers, Sharad Goel

**Affiliations:** ^1^ Applied Statistics, Social Science, and Humanities, New York University, New York, NY 10012, USA.; ^2^ Harvard Kennedy School, Harvard University, Cambridge, MA 02138, USA.; ^3^ Stanford Law School, Stanford University, Stanford, CA 94305, USA.; ^4^ Computer Science, Stanford University, Stanford, CA 94305, USA.

## Abstract

Millions of Americans must attend mandatory court dates every year. To boost appearance rates, jurisdictions nationwide are increasingly turning to automated reminders. However, previous research paints an incomplete picture of their effectiveness—in particular, there has been little work assessing the impact of reminders on downstream arrests and incarceration. In partnership with the Santa Clara County Public Defender’s Office, we randomly assigned 5709 public defender clients to either receive automated text message reminders (treatment) or not receive reminders (control). We found that reminders reduced warrants issued for missed court dates by ~20%, with 12.1% of clients in control issued a warrant compared to 9.7% of clients in treatment. Further, we found that incarceration from missed court dates dropped by a similar amount, from 6.6% in control to 5.2% in treatment. The effectiveness of reminders bolsters the theory that lapses in memory or comprehension contribute to missed court appearances.

## Teaser

Automated text message reminders reduce pretrial incarceration for missed court dates among public defender clients.

## INTRODUCTION

In the United States, after a person is arrested and charged with a crime, they are either held in jail as their case proceeds or they are released and asked to attend court of their own accord. While many released defendants do indeed attend court—as is legally required—some fail to do so. Failing to appear (FTA) at a required court date is a crime in nearly every state (*1*). FTA rates typically range from 10 to 20% per court date, with some jurisdictions reporting rates as high as 50% per court date (*2*, *3*). FTAs create inefficiency for the court system more broadly, increasing costs and exacerbating delays in US courts (*4*).

An FTA can prompt judges to issue a warrant mandating the defendant’s arrest—hereafter called a “bench warrant”—at their next encounter with law enforcement. Judges often issue a bench warrant when a defendant does not attend a mandatory court date, although in some states they can decline to do so if they believe the client has sufficient justification for not being present (e.g., due to sickness). Although FTA itself is a minor infraction, bench warrants are the most common open warrant type across much of the United States (*5*–*8*).

Once a person is arrested on a bench warrant, they are often held in jail to guarantee their appearance in court. Arrests on open bench warrants are the most common reason for jail admission in many American jurisdictions, comprising 14 to 30% of all bookings (*9*–*14*). This pretrial incarceration can impose social and economic hardships on defendants and their families—including housing loss, family strain, and social stigma (*15*, *16*)—even as evidence suggests that time in jail may not deter future court absences (*17*). Pretrial incarceration has also been found to increase recidivism and reduce employment and wealth (*18*–*22*). The consequences of missed court dates may fall particularly hard on racial minorities, given the disproportionate involvement of marginalized communities in the criminal legal system (*5*, *16*, *23*, *24*). Missed court dates likely result from a combination of intentional noncompliance, logistical challenges (e.g., issues arranging work schedules, finding childcare, or securing transportation to court), and forgetfulness (*25*–*28*). This last hypothesis—forgetfulness—mirrors established patterns of human behavior in other domains, including health care, finance, politics, and education (*29*–*38*). Court date reminders are intended to mitigate this forgetfulness and help people plan for upcoming court obligations (*39*).

Nearly half of all counties nationwide have implemented or plan to implement court date reminders via text message, phone call, mail, or some other method (*40*). Studies have shown that text message reminders can be effective for nondefendant participants in the criminal legal system (*41*, *42*). For example, an experiment in Arkansas found that text message reminders reduced missed probation and parole appointments by more than 40% (*42*). There are also several studies on the effectiveness of reminders sent to defendants via resource-intensive methods like a letter or a telephone call (*43*–*51*). One such study found that postcard reminders reduced nonappearance rates by up to 34% in an experiment with misdemeanor defendants in Nebraska (*51*) [see (*52*, *53*) for reviews of the relevant literature]. Yet, research on the effects of automated text message reminders is limited, although it is one of the most cost-effective ways of sending reminders, and is increasingly used by jurisdictions nationwide.

Previous randomized controlled trials (RCTs) paint an incomplete picture of the efficacy of text message reminders to increase court appearance rates and decrease the negative consequences of missing court (Table 1). Two recent RCTs found statistically significant and meaningful reductions in FTA rates from text message reminders (*54*, *55*), while two other RCTs also found reductions in FTA rates, although the estimates were not statistically significant (*3*, *56*). Another RCT estimated higher—but not statistically significant—warrant rates among people who received a text message reminder (*57*). In addition, we note that the two studies that found statistically significant impacts on FTA considered only municipal violations and misdemeanors, although more serious felony-level cases often comprise a substantial proportion of a court’s caseload. Only one of the above studies examined the impact of automated reminders on pretrial incarceration but did not find a statistically significant effect on jail bookings (*54*). This missing link in the literature raises questions about whether automated court date reminders achieve any social benefits beyond increased adherence to court date schedules.

**Table 1.** Inconsistent historical evidence on the efficacy of automated court date reminders. Past experiments have yielded mixed results on the effectiveness of automated text message reminders for improving appearance rates. No study has established a connection between automated text message reminders and jail incarceration. N/A, not available.

Study	Year	Outcome	Sample	Ctrl rate	Est. effect	95% CI	Est. rel. effect	*P* value
Chivers and Barnes, 2018 (*57*)	2017	Warrant at court date	946 defendants	22.5%	+1.8 pp	N/A	+8%	0.51
Lowenkamp *et al.*, 2018 (*58*)	N/A	FTA at court date	10,228 defendants	13%	−2 pp	N/A	−18%	0.07
Fishbane *et al.*, 2019 (*55*)	2016–2017	FTA/warrant at summons hearing	20,234 defendants	37.9%	9.9 pp	[−12–7.8 pp]	−26%	<0.01
Emanuel and Ho, 2024 (*54*)	2018–2019	FTA at arraignment	30,870 defendants	21%	−8.2 pp	N/A	−39%	<0.01
Owens and Sloan, 2023 (*3*)	2021	FTA at court date	1,096 housed defendants	50%	–6 pp	[−11.2–+0.6 pp]	−12%	0.08

To help resolve whether text message reminders increase court appearance and reduce incarceration, we ran an RCT with clients at the Santa Clara County Public Defender Office (SCCPDO), headquartered in San Jose, California. Our experiment consists of 5709 SCCPDO clients who were charged with felonies, misdemeanors, or supervision violations. Of these clients, 2387 had court dates between 17 May 2022 and 21 September 2022, and 3322 had court dates between 14 October 2022 and 24 August 2023. Clients were randomly assigned to treatment or control conditions with equal probability: 2811 clients were assigned to the control condition, which meant they did not receive any automated reminders; and 2898 clients were assigned to the treatment condition. To be eligible for inclusion in the experiment, clients must have had at least one court date in the timespans mentioned above, had a cellphone number available in SCCPDO’s case management system, and had never previously received an automated reminder from SCCPDO.

**Fig. 1.**
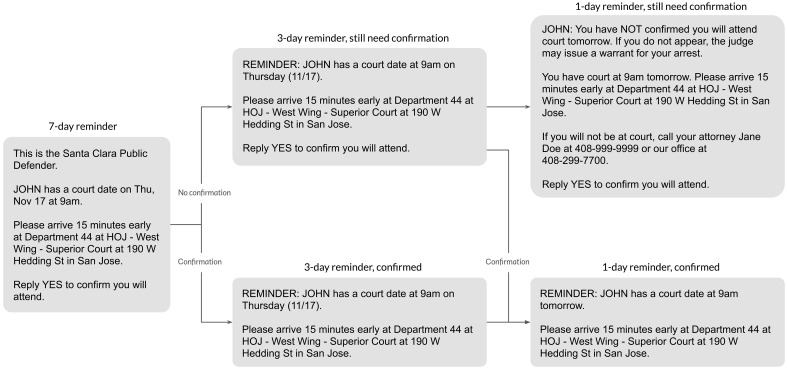
Message flow for clients in the treatment condition. Clients were asked to confirm their attendance at each court date, with the timing of their confirmation determining their path through this flow. Reminders were also available in Spanish and Vietnamese for clients who required translation in these languages (fig. S2).

**Fig. 2.**
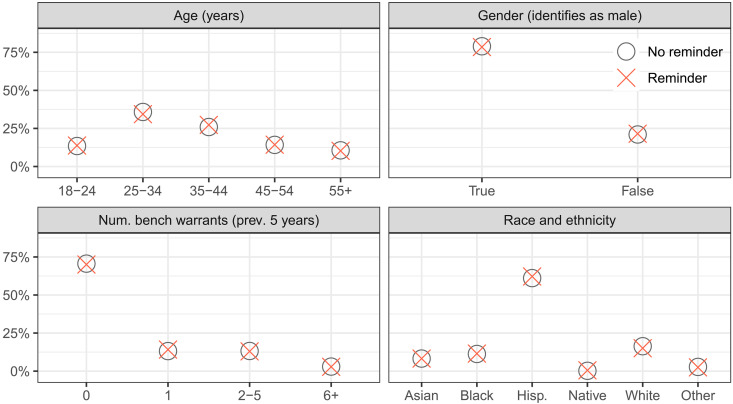
Distributions of select covariates split between treatment and control populations. Nearly half of participants were under the age of 34, most identified as male, a majority identified as Hispanic, and most had no record of a bench warrant within the past 5 years. In addition, distributions are nearly identical between conditions, confirming that our assignment mechanism randomly assigned clients to the two conditions as intended. See fig. S1 for an expanded version.

Clients in the treatment condition received reminders 7 days, 3 days, and 1 day before each upcoming court date (see Fig. 1 for a diagram of these reminders). The reminder schedule we used mirrors the timing of reminders in other studies, which sent reminders at various combinations of 1, 3, and 7 days before a court date (*47*, *52*, *54*, *55*, *57*). Translated versions of these reminders were provided in Spanish and Vietnamese for the 22% of clients who had previously indicated a need for translation in these languages (fig. S2). In Fig. 2 and fig. S1, we show that covariate distributions were nearly identical across experiment arms, indicating that the randomization scheme worked as intended.

Our study is the first to specifically examine the effect of reminders for clients of public defenders. Understanding the efficacy of reminders for this subpopulation is particularly important for ongoing policy debates, as policymakers may expect that public defenders can ensure court appearance for their own clients, obviating the need for reminders sent at additional cost to taxpayers. Indeed, SCCPDO clients appear at their court appointments the vast majority of the time. Yet, there is still room for improvement, with about 10 to 15% of scheduled court dates for SCCPDO clients ending in a bench warrant for nonappearance. Given that people are often required to attend multiple court dates, nearly one-third of SCCPDO clients received at least one bench warrant for missing court over the course of 2022. Over half of these clients were only facing misdemeanor or lesser charges, and one of every four had no history of prior charges on file with SCCPDO. A single bench warrant for these clients thus has the potential to quickly ramp up an otherwise minimal brush with the criminal legal system and underscores the importance of increasing appearance rates.

## RESULTS

We focus on how reminders affect two primary outcomes: (i) issuance of a bench warrant for failure-to-appear (FTA) and (2) remands to custody on a bench warrant. When measuring impacts on bench warrant issuance, we consider two timeframes: first, issuance at the client’s first scheduled court date after assignment, and second, issuance of at least one bench warrant at any point between assignment and the end of the experiment. When measuring impacts on remands to custody, we solely consider the second (longer) timeframe, since several events must transpire before a remand occurs: First, a client must miss court, then a judge must issue a bench warrant, and last, sometime later, the defendant must have a chance encounter with law enforcement that results in an arrest for the open warrant.

In the control condition, 12.1% of clients received a bench warrant at their first scheduled court date after assignment compared to 9.7% for clients in the treatment condition. This 2.5 percentage point (pp) difference [95% confidence interval (CI): 0.9 to 4.1 pp] corresponds to a 20.4% reduction in bench warrant rates. Between assignment and the end of the experiment, 20.7% of clients in the control condition received at least one bench warrant compared to 17.6% of clients in the treatment condition, a 3.1 pp difference (95% CI: 1.1 to 5.1 pp), corresponding to a 15.0% reduction in the issuance of bench warrants.

These reductions persist in subsequent rates of incarceration. A total of 6.6% of clients in the control condition were remanded on a bench warrant after assignment to our experiment compared to 5.2% of clients in the treatment condition, a 1.4 pp difference (95% CI: 0.2 to 2.6 pp) corresponding to a relative reduction of 21.4%. Impacts on both bench warrants and remands to custody are qualitatively similar for clients with both misdemeanor- and felony-level cases, suggesting that both groups benefit from automated reminders (table S3).

To improve the precision of our results, we also estimate the impact of text message reminders via logistic regression models corresponding to each of our outcomes of interest, adjusting for available pretreatment covariates (see the “Statistical analysis” section). With these models, we estimate effects that are comparable to those seen with the raw, unadjusted rates (Table 2).

**Table 2.** The effect of text message reminders on the issuance of bench warrants for nonappearance and on remands to custody on a bench warrant. These effects were estimated using logistic regression (see the “Statistical analysis” section for more information). Reported estimates are odds ratios (i.e., exponentiated logistic regression coefficients), with SEs in parentheses calculated using the delta method. The single star indicates that the corresponding logistic regression coefficient estimates (on the log-odds scale) have a *P* value between 0.01 and 0.05, and the double star indicates a *P* value between 0.001 and 0.01. Coefficient estimates for other covariates are included in table S2.

Timeframe	First court date			Any court date		
**Outcome**	**Bench warrant**				**Remand to custody**	
	**(1)**	**(2)**	**(3)**	**(4)**	**(5)**	**(6)**
Observations	5709 clients		5709 clients		5709 clients	
Obs. (control)	2811 clients		2811 clients		2811 clients	
Obs. (treatment)	2898 clients		2898 clients		2898 clients	
Rate (control)	12.1%		20.7%		6.6%	
Rate (treatment)	9.7%		17.6%		5.2%	
Difference	−2.5 pp		−3.1 pp		−1.4 pp	
Est. treat. effect	0.748**	0.775**	0.797**	0.818**	0.768*	0.775*
SE	(0.068)	(0.066)	(0.058)	(0.055)	(0.094)	(0.088)
Covar. adjustment	Yes	No	Yes	No	Yes	No

## DISCUSSION

We found that automated reminders increased court attendance and reduced pretrial incarceration among public defender clients by ~20%. Given the large number of people who are arrested and detained for missing court each year, pretrial reminders could yield substantial societal benefits (*18*, *58*). The effectiveness of these simple behavioral nudges suggests that forgetfulness or lack of comprehension is an important factor explaining missed court dates. Our results highlight the value of addressing cognitive and informational barriers to court attendance instead of relying solely on punitive measures to ensure attendance. Further, with an average marginal cost of ~60¢ per defendant per case, automated text message reminders can be an effective and relatively inexpensive way to help people attend court and avoid incarceration.

Building on our results, further research could help optimize these behavioral nudges to maximize court appearances. For example, the optimal timing and frequency of text message reminders is unclear. It may be more effective to remind clients about court obligations over a week in advance or to do so more frequently in the week before. The reminders we used also only briefly mentioned the possible consequences of missing court. Other content—a stronger focus on the consequences or a focus on possible supports—may be more effective at preventing bench warrants. In addition, court date reminders may not help clients who are struggling with more fundamental barriers to court attendance, such as lack of transportation or childcare or work obligations. Transportation or financial assistance might further address these barriers and could complement court date reminders (*59*). Relatedly, many public defender clients may lack a reliable and persistent cellphone number altogether, preventing them from benefiting from these types of interventions. Other court participants (including witnesses and police officers) may also struggle to attend court and may benefit from reminders such as the ones we describe here (*60*). The interaction between reminders and other interventions also calls for additional research. For example, many pretrial risk assessment algorithms were trained to predict nonappearance risk using a sample of historical court dates that did not benefit from court date reminders. As a result, these tools may overestimate risk for people receiving reminders, underscoring the need for alternative risk assessment models that can account for these interventions (*61*).

In addition to behavioral nudges, policymakers might consider alternative pathways to reducing pretrial incarceration. For example, judges could issue a bench warrant for nonappearance only in the most egregious circumstances, such as when there is clear evidence a defendant is unwilling to cooperate with the judicial process. Some counties in California are working to improve appearance rates and other outcomes by pairing defendants with case managers that help to address underlying challenges, such as housing instability and substance use, which their clients may be facing. Also, courts may consider alternative means to case resolution that do not require the hassle of physical appearance in court, e.g., participation in ongoing proceedings via asynchronous online portals for less serious cases (*62*). Despite the potential of behavioral nudges, it is also important to understand their limitations. In our setting, reminders would likely have a small impact on overall jail populations, as incarceration for FTA is typically shorter than other offenses (*9*). Accordingly, we view behavioral nudges as just one step toward broadly improving the criminal legal system.

## MATERIALS AND METHODS

### Experimental design

For clients in the treatment condition, we sent an introductory text message before the first reminder explaining the reminder program and explaining how to opt out, if desired. Of the 2898 clients in the treatment arm, 107 opted out of receiving text message reminders, of which a majority (57 clients) opted out by noting that we had the wrong number. After a reminder, clients were prompted to confirm their attendance by responding with “yes” or similar affirmations. For example, our application recognized many possible confirmations, including “OK,” “Confirmed,” “I’ll be there”, a thumbs-up emoji, and confirmations in Spanish (such as Sí or Gracias) and Vietnamese (like Đi or Được). If they confirmed, we did not prompt for confirmation on subsequent reminders. Ultimately, 51% of clients in the treatment arm confirmed their attendance, and among these clients, 2.9% received a bench warrant at their first court date; in comparison, a bench warrant was issued for 16.8% of clients who did not confirm their attendance (table S4). This difference could be explained by the act of confirming, self-selection, or a combination thereof. In any case, our study is not designed to determine whether confirmations affect appearance in court, and we do not consider confirmation behavior in our analysis to avoid posttreatment bias.

In our original preregistration for this experiment (available at https://aspredicted.org/SMY_N1R), we proposed comparing two message variants: a variant drafted by the public defender versus a simpler variant that we thought would be easier for clients to understand (*63*). However, we later concluded that the two message variants were not meaningfully comparable (e.g., because they differed in length) and so terminated that experiment without analyzing any of the resulting data. Simultaneously, we ran the experiment described in this paper, comparing the simpler variant against no messages. For transparency, we have now analyzed the data corresponding to our two-variant experiment, finding that bench warrant rates were lower among clients receiving the simpler variant compared to the longer one (12.3% versus 13.4%, respectively), although the difference was not statistically significant. We are currently running an experiment that is better designed to compare message templates, preregistered at https://aspredicted.org/FKC_XYY.

### Statistical analysis

Bench warrants are rarely proactively enforced. In theory, clients may resolve an open bench warrant by independently reaching out to the court or their attorney. In practice, however, clients with open bench warrants are typically arrested during their next, unrelated encounter with law enforcement (e.g., as a result of a traffic stop). In either case, the client then appears at a “bench warrant hearing.” At these hearings, judges may choose to release the client back into the community if they believe the client will appear at future court dates or—alternatively—may remand the client to jail pending bail, later release, or case resolution. As a result, a bench warrant does not always result in incarceration, given the dynamics of client initiative, chance contact with law enforcement, and judicial discretion. In particular, only half of SCCPDO clients with bench warrants issued between 2019 and 2021 were remanded to jail within 2 years of their missed court date.

We are specifically interested in how court date reminders affect these remands to jail. To measure this phenomenon, we code a client’s outcome as “incarcerated” if they were remanded at a bench warrant hearing where no new charges were brought and code the outcome as “not incarcerated” for all other clients. This metric directly corresponds to the target of our intervention—incarceration solely attributable to missed court dates (discussed in more detail in S2). Our findings are qualitatively similar if we redefine the outcome to indicate whether a client was remanded at any type of bench warrant hearing, regardless of whether they were arrested on new charges.

In Table 2, we estimate the impact of text message reminders using logistic regression models of the following form

$$\text{Pr}\left(Y_{i}=1\right)={\text{logit}}^{-1}\left(\alpha +\beta T_{i}+\gamma^{T}X_{i}\right)$$ (1)

where $Y_{i}$ indicates our outcome of interest (e.g., issuance of a bench warrant); $T_{i}$ indicates whether the client was in the treatment condition; and $X_{i}$ is a vector representing a variety of observable features of the client, case, and first scheduled court date. In particular, $X_{i}$ includes demographic information (the client’s age, race, whether the client identifies as male, whether the client prefers a language interpreter, whether the client’s attorney indicated a possible mental health issue for the client, whether a home address is on file for the client, and the distance between the client’s home address and the courthouse where their appearance is scheduled, coded as zero if there is no address on file), client history [the number of bench warrants for nonappearance known to SCCPDO in the previous 5 years, the inverse number of court dates known to SCCPDO in the previous 5 years, the product of these two covariates, representing the client’s bench warrant rate for FTA over the past 5 years, whether the client was “new,” i.e., whether the earliest court date known to the public defender was in the preceding year, the number of previous cases with the public defender’s office, the number of previous convictions or guilty pleas with the public defender office (including nolo contendere pleas), and the number of years since the client’s phone records were updated], case information (whether the most serious charge was classified as a felony, misdemeanor, or supervision violation and indicators for which of 19 high-level charge categories were present, e.g., burglary or robbery), and court date information (the courthouse where the court date was scheduled, the day of week, the month, and a number indicating the court date was the *n*-th scheduled appointment on a case).
