# Supplementary material for: Automated reminders reduce incarceration for missed court dates: Evidence from a text message experiment
Source: Sci Adv. 2025 Oct 1;11(40):eadx7483. doi: 10.1126/sciadv.adx7483 (PMC12487872; doi:10.1126/sciadv.adx7483)
Supplement: Supplementary file 1 — Supplementary Text S1 and S2 Figs. S1 and S2 Tables S1 to S4 [file sciadv.adx7483_sm.pdf]

Science Advances
AAAS

Supplementary Materials for

Automated reminders reduce incarceration for missed court dates: Evidence from a text message experiment

Alex Chohlas-Wood *et al.*

Corresponding author: Alex Chohlas-Wood, alex.cw@nyu.edu

Sci. Adv. **11**, eadx7483 (2025)
DOI: 10.1126/sciadv.adx7483

This PDF file includes:

Supplementary Text S1 and S2
Figs. S1 and S2
Tables S1 to S4

S1 Treatment assignment

Starting in 2021, as we developed the software necessary to conduct this experiment, we sent court date reminders to some SCCPDO clients; these clients were not eligible for inclusion in our experiment, though they received similar reminders to those described here.

In the first phase of the experiment (i.e., for clients with initial court dates between May 17, 2022 and September 21, 2022), clients in the treatment condition received an introductory text message up to seven days before their first court date reminder. Occasionally, however, court dates once eligible for reminders may have become ineligible in this interim period after the introductory message was sent (e.g., because the attorney indicated they would appear on the client's behalf, or because the recipient may have opted out of text message reminders immediately after their introductory message). As a result, 85 of the 2,898 clients in the treatment condition did not receive a reminder for their initially scheduled court date. Nevertheless, we include in the treatment condition all clients who received an introductory message, regardless of whether a reminder was actually sent, as the introductory text message could itself impact behavior. In the second phase of the experiment (i.e., for clients with initial court dates between October 14, 2022 and August 24, 2023), we adjusted our protocol to address this issue, sending both the introductory message and the first court date reminder at the same time. This change ensures that all clients in the treatment condition did in fact receive at least one reminder.

At the end of the first phase of the experiment, all clients in the first phase were transitioned to receive text messages reminders for any future court dates, regardless of whether they were initially assigned to treatment or control. As a result, our estimate of the effect of reminders on long-term outcomes is likely conservative, since some clients in the control condition received reminders for part of the observation window. This pattern does not affect our estimate of reminders on the issuance of bench warrants at the first court date, since that outcome is measured before any transitioning occurred. No clients in the second phase of the experiment were transitioned, i.e., clients in the control condition in the second phase did not receive reminders during the observation period.

To confirm that our assignment procedure indeed randomly assigned clients to treatment or control, we examined balance plots (Table S1 and Figure S1). Across a wide range of covariates, we see that the distributions are nearly identical between the two conditions, as expected.

S2 Verifying custody status

To verify that a client who was remanded to custody was, in fact, held in the county jail, we worked with SCCPDO to manually query the custody status for a sample of 41 clients with court dates between June 5 and June 7, 2023. Of these 41 clients, 19 were remanded to custody at their court date. As of June 9, 2023, 16 of these 19 clients remanded to custody were verified to be in jail. Of the remaining 22 clients (who were not remanded to custody), 21 were verified not to be in jail. The small discrepancy between remands and incarceration is likely due to events that transpired between the court date and the custody check; for example, clients may have been released after paying bail, or may have been incarcerated on a different case not represented by SCCPDO. These results suggest that the vast majority of clients remanded to custody spend at least several days in jail.

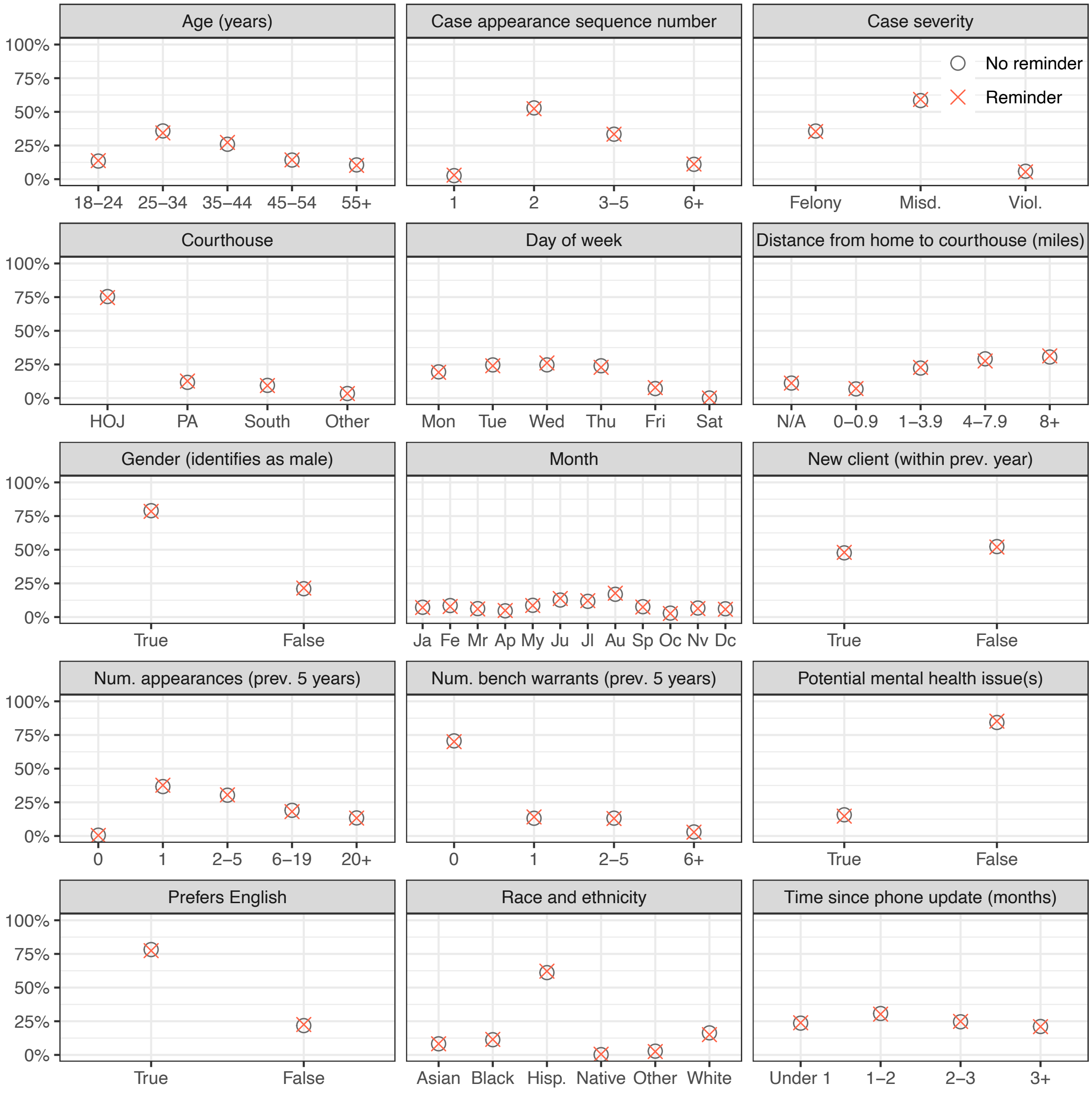

Figure S1: Covariate distributions for the treatment and control conditions were nearly identical, confirming that our assignment mechanism correctly randomly assigned clients to the two conditions. Statistics in this figure are drawn from the last two columns in Table S1.

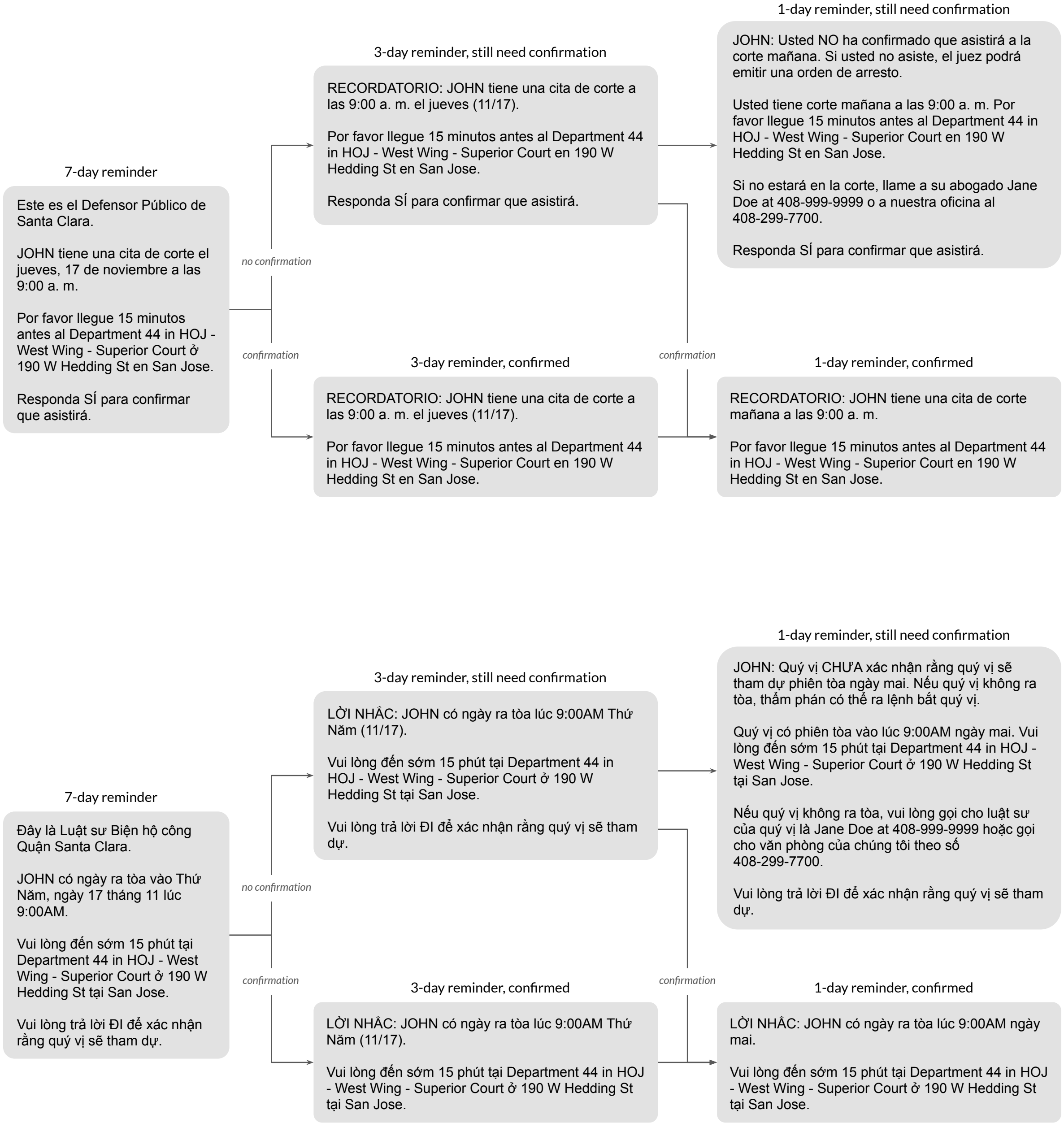

Figure S2: Reminder flows in Spanish (top) and Vietnamese (bottom).

	All	Cell on File	Experiment	Control	Treatment
**Age (years)**					
18-24	12%	13%	14%	13%	14%
25-34	33%	34%	35%	36%	34%
35-44	27%	28%	27%	26%	27%
45-54	15%	15%	14%	14%	14%
55+	13%	10%	10%	11%	10%
**Any bench warrant remand (without new charges) during experiment**					
True	3%	11%	6%	7%	5%
False	97%	89%	94%	93%	95%
**Any bench warrant remand during experiment**					
True	3%	13%	7%	8%	6%
False	97%	87%	93%	92%	94%
**Any remand during experiment**					
True	26%	34%	23%	24%	22%
False	74%	66%	77%	76%	78%
**Case appearance sequence number**					
1	5%	36%	3%	3%	3%
2	30%	16%	53%	53%	52%
3-5	32%	22%	33%	33%	33%
6+	33%	25%	11%	11%	11%
**Case severity**					
Felony	35%	37%	36%	36%	35%
Misdemeanor	49%	57%	59%	58%	59%
Supervision Violation	16%	6%	6%	6%	5%
**Courthouse**					
Hall Of Justice	67%	70%	75%	75%	75%

Palo Alto	12%	10%	12%	12%	13%
South County	7%	8%	9%	9%	9%
Other	14%	11%	3%	3%	3%
**Distance from home to courthouse (miles)**					
0-0.9	6%	7%	7%	7%	7%
1-3.9	19%	22%	23%	22%	23%
4-7.9	24%	28%	28%	29%	28%
8+	26%	29%	31%	31%	31%
N/A	25%	14%	11%	11%	11%
**Identifies as male**					
True	80%	80%	79%	79%	78%
False	20%	20%	21%	21%	22%
**New client (within prev. year)**					
True	34%	38%	48%	48%	48%
False	66%	62%	52%	52%	52%
**Num. appearances (prev. 5 years)**					
0	4%	23%	0.5%	0.6%	0.4%
1	20%	11%	37%	37%	38%
2-5	26%	21%	30%	30%	31%
6-19	28%	24%	19%	19%	18%
20+	23%	22%	13%	13%	13%
**Num. bench warrants (prev. 5 years)**					
0	61%	64%	70%	71%	70%
1	15%	13%	14%	13%	14%
2-5	19%	17%	13%	13%	13%
6+	5%	5%	3%	3%	3%
**Potential mental health issue(s)**					
True	16%	18%	15%	16%	15%

False	84%	82%	85%	84%	85%
**Prefers English**					
True	82%	80%	78%	78%	77%
False	18%	20%	22%	22%	23%
**Race and ethnicity**					
Asian	8%	8%	8%	8%	8%
Black	11%	12%	11%	11%	12%
Hispanic	55%	60%	62%	61%	62%
Native	0.3%	0.4%	0.4%	0%	0.5%
White	17%	17%	16%	16%	15%
Other	8%	3%	3%	3%	2%
**Total clients**	**21,343**	**14,063**	**5,709**	**2,811**	**2,898**

Table S1: Population characteristics for five subsets of SCCPDO clients. The first population, "all" clients, was created by considering all SCCPDO clients with a reminder-eligible court date during the experiment window and measuring client and case characteristics at the first reminder-eligible court date for each client within this window. The second population, "cell on file", was constructed by considering all SCCPDO clients who had a cellphone number on file and a reminder-eligible court date during the experiment window, and measuring attributes at the first reminder-eligible court date for each client within the experiment window. The third population, "experiment" clients, represents all clients in the experiment population at their first observed court date. Clients in the "cell on file" population who are not in the "experiment" population were not eligible for our experiment because they received reminders before our experiment began. The fourth and fifth populations, "treatment" clients and "control" clients, further break down the "experiment" population by the client's random assignment.

Timeframe	First Court Date	Any Court Date	
Outcome	Bench Warrant		Incarceration
Model	(1)	(3)	(5)
**Intercept**			
-	-2.54*** (0.73)	-1.17* (0.58)	-2.66** (0.83)
**Treatment**			
Reminders	-0.29** (0.09)	-0.23** (0.07)	-0.26* (0.12)
**Client race/ethnicity**			
Asian	-0.26 (0.19)	-0.05 (0.16)	0.15 (0.26)
Black	-0.35* (0.17)	-0.07 (0.14)	0.12 (0.22)
Hispanic	-0.23 (0.13)	-0.21* (0.11)	-0.05 (0.17)
Native	0.57 (0.59)	0.06 (0.54)	0.93 (0.78)
Other	-0.61 (0.36)	-0.12 (0.25)	-0.17 (0.46)
**Client information**			
Is not male	-0.18 (0.12)	-0.07 (0.09)	-0.18 (0.17)
Age	0.00 (0.00)	0.00 (0.00)	-0.01 (0.01)
Mental health	-0.03 (0.13)	-0.05 (0.10)	0.22 (0.15)
New client	-0.07 (0.13)	0.10 (0.11)	0.05 (0.21)
Prefers english	-0.05 (0.13)	0.06 (0.10)	0.19 (0.18)
Years since phone added	0.54*** (0.11)	0.36*** (0.09)	-0.06 (0.14)
Home address recorded	-0.61*** (0.12)	-0.59*** (0.10)	-0.61*** (0.15)
Miles from home to court	0.00 (0.00)	0.00 (0.00)	0.00 (0.00)
**Client history (5 yr counts)**			
Cases	0.02* (0.01)	0.02* (0.01)	0.02 (0.01)
Convictions	0.00 (0.00)	0.00 (0.00)	0.00 (0.01)
1/court dates	-0.05 (0.37)	-0.55 (0.31)	-2.83*** (0.63)
Bench warrants	0.01 (0.03)	0.04 (0.03)	0.07 (0.03)

Bench warrants/court dates	2.87*** (0.45)	3.07*** (0.39)	2.99*** (0.61)
**Day of week**			
Monday	0.64** (0.23)	0.32 (0.17)	0.25 (0.27)
Tuesday	0.45* (0.22)	0.27 (0.16)	0.13 (0.26)
Wednesday	0.64** (0.22)	0.32* (0.16)	0.33 (0.25)
Thursday	0.48* (0.22)	0.24 (0.16)	0.05 (0.27)
**Month**			
February	-0.12 (0.24)	0.00 (0.18)	0.06 (0.36)
March	0.10 (0.25)	0.04 (0.19)	-0.25 (0.42)
April	0.29 (0.26)	-0.12 (0.21)	-0.25 (0.46)
May	0.14 (0.23)	-0.16 (0.18)	0.35 (0.33)
June	0.21 (0.21)	-0.09 (0.16)	0.63* (0.30)
July	-0.10 (0.22)	-0.50** (0.17)	0.45 (0.31)
August	0.05 (0.21)	-0.90*** (0.17)	0.27 (0.30)
September	0.37 (0.23)	-0.68*** (0.20)	0.28 (0.35)
October	0.40 (0.30)	0.42 (0.23)	0.77 (0.39)
November	0.07 (0.24)	0.21 (0.18)	0.38 (0.35)
December	0.06 (0.25)	-0.03 (0.19)	-0.11 (0.39)
**Court date info**			
Appearance number	-0.01 (0.01)	-0.01 (0.01)	-0.03 (0.01)
**Case severity**			
Felony	-0.22 (0.23)	-0.07 (0.18)	0.01 (0.24)
Misdemeanor	0.24 (0.22)	0.30 (0.18)	0.02 (0.23)
Prcs violation	1.68 (1.27)	0.05 (0.66)	0.24 (0.89)
Probation violation	1.73 (1.26)	0.06 (0.61)	0.64 (0.85)
**Courthouse**			
Hall Of Justice	0.65 (0.61)	0.31 (0.49)	0.16 (0.65)
Family Court	-1.02 (0.75)	0.24 (0.53)	0.54 (0.70)
Palo Alto	0.19 (0.62)	-0.25 (0.50)	0.02 (0.67)

San Jose Municipal	-0.04 (0.46)	0.42 (0.34)	-0.17 (0.62)
Morgan Hill	0.16 (0.63)	-0.35 (0.51)	0.23 (0.68)
Other	0.40 (0.37)	0.28 (0.33)	0.81 (0.44)
**Charges**			
Assault	-0.34** (0.12)	-0.19 (0.10)	0.32* (0.15)
Burglary	0.22 (0.25)	0.27 (0.20)	0.37 (0.27)
Disorderly	-0.15 (0.48)	-0.09 (0.39)	0.99* (0.45)
Driving	-0.45*** (0.13)	-0.22* (0.11)	-0.61** (0.20)
Drugs	0.54*** (0.13)	0.56*** (0.12)	0.44* (0.18)
Forgery	-0.43 (0.59)	-0.06 (0.47)	-1.83 (1.12)
Fraud	0.56** (0.18)	0.40* (0.16)	-0.10 (0.28)
Homicide	-1.51 (1.01)	-1.09 (0.60)	-0.66 (1.03)
Kidnapping	-1.17 (0.60)	-0.87* (0.39)	-0.44 (0.55)
Larceny	0.39* (0.16)	0.53*** (0.13)	0.33 (0.20)
Larceny (vehicular)	0.86*** (0.20)	0.89*** (0.18)	0.79** (0.25)
Probation/parole	-2.20 (1.27)	-0.28 (0.61)	-0.15 (0.85)
Robbery	0.01 (0.36)	-0.13 (0.27)	-0.47 (0.44)
Sex offenses	-1.12* (0.47)	-1.39*** (0.40)	-1.26 (0.73)
Stolen property	0.61* (0.24)	0.75*** (0.22)	0.01 (0.32)
Trespassing	0.58* (0.26)	0.27 (0.23)	0.48 (0.32)
Weapons	-0.17 (0.18)	-0.08 (0.15)	0.08 (0.23)
Vandalism	0.27 (0.18)	0.30* (0.14)	0.31 (0.20)
Other	-0.14 (0.12)	-0.04 (0.10)	0.30* (0.14)

Table S2: Logistic regression coefficient estimates for the three covariate-adjusted models (see Results in the main paper). Model numbers correspond to those listed in Table 2. Coefficient estimates are on the log-odds scale, with standard errors in parentheses. A single star indicates that the corresponding logistic regression coefficient estimate has a p-value between 0.01 and 0.05, a double star indicates a p-value between 0.001 and 0.01, and a triple star indicates p-values under 0.001.

Timeframe	First Court Date	Any Court Date	
Outcome	Bench Warrant		Incarceration
Model	(7)	(8)	(9)
**Intercept**			
Intercept	-1.96*** (0.07)	-1.35*** (0.06)	-2.87*** (0.10)
**Treatment**			
Reminders	-0.25* (0.11)	-0.20* (0.08)	-0.19 (0.15)
**Case severity**			
Felony	-0.06 (0.12)	0.03 (0.10)	0.53*** (0.15)
**Interaction**			
Reminders * Felony	-0.01 (0.18)	-0.01 (0.14)	-0.14 (0.23)

Table S3: Logistic regression coefficient estimates for three alternate models that include an interaction between the treatment and case severity. Reminder impacts on bench warrant rates appear qualitatively similar for both felony-level and misdemeanor-level clients. The model estimate of reminders' impact on incarceration rates for felony-level clients is negative but highly uncertain, a result of the fact that this experiment is not powered to determine whether reminders are more effective for clients with felony charges compared to those with less serious cases. Coefficient estimates are on the log-odds scale, with standard errors in parentheses. A single star indicates that the corresponding logistic regression coefficient estimate has a p-value between 0.01 and 0.05, a double star indicates a p-value between 0.001 and 0.01, and a triple star indicates p-values under 0.001.

Confirmed?	Proportion of Clients	Bench Warrant Rate
Yes	51%	2.9%
No	49%	16.8%

Table S4: Proportion of clients in treatment arm who confirmed and did not confirm, and their corresponding bench warrant rates at the first observed court date. Note that confirmation behavior is a response to the reminder, and as such the act of confirmation cannot be interpreted causally.
